# Subcutaneous injection of adipose stromal cell-secretome improves renal function and reduces inflammation in established acute kidney injury

**DOI:** 10.1186/s13287-024-03736-x

**Published:** 2024-04-24

**Authors:** Md Mahbub Ullah, Jason A. Collett, Jacob C. Monroe, Dmitry Traktuev, Michael Coleman, Keith L. March, David P. Basile

**Affiliations:** 1 Department of Anatomy, Cell Biology & Physiology, Indiana University School of Medicine, 635 Barnhill Dr. MS 2063, Indianapolis, IN 46202 USA; 2 Division of Cardiovascular Medicine and Center for Regenerative Medicine, University of Florida, Gainesville, FL USA; 3 Theratome Bio, Inc., Indianapolis, IN USA

**Keywords:** AKI, CKD, Th17 cells, Macrophages, Kidney repair

## Abstract

**Background:**

Adipose stromal cells (ASC) are a form of mesenchymal stromal cells that elicit effects primarily via secreted factors, which may have advantages for the treatment of injury or disease. Several previous studies have demonstrated a protective role for MSC/ASC on mitigating acute kidney injury but whether ASC derived factors could hasten recovery from established injury has not been evaluated.

**Methods:**

We generated a concentrated secretome (CS) of human ASC under well-defined conditions and evaluated its ability to improve the recovery of renal function in a preclinical model of acute kidney injury (AKI) in rats. 24 h following bilateral ischemia/reperfusion (I/R), rats were randomized following determination of plasma creatinine into groups receiving vehicle -control or ASC-CS treatment by subcutaneous injection (2 mg protein/kg) and monitored for evaluation of renal function, structure and inflammation.

**Results:**

Renal function, assessed by plasma creatinine levels, recovered faster in ASC-CS treated rats vs vehicle. The most prominent difference between the ASC-CS treated vs vehicle was observed in rats with the most severe degree of initial injury (P_cr_ > 3.0 mg/dl 24 h post I/R), whereas rats with less severe injury (P_cr_ < 2.9 mg/dl) recovered quickly regardless of treatment. The quicker recovery of ASC-treated rats with severe injury was associated with less tissue damage, inflammation, and lower plasma angiopoietin 2. In vitro, ASC-CS attenuated the activation of the Th17 phenotype in lymphocytes isolated from injured kidneys.

**Conclusions:**

Taken together, these data suggest that ASC-CS represents a potent therapeutic option to improve established AKI.

**Supplementary Information:**

The online version contains supplementary material available at 10.1186/s13287-024-03736-x.

## Background

Acute kidney injury (AKI) remains a major therapeutic challenge and economic burden to the healthcare system [1]. It is estimated that 1 in 5 hospitalized patients may develop AKI, and this is associated with a 20–25% mortality rate and an increased hospitalization cost estimated between $5.4 to $24 billion USD [2]. Moreover, AKI strongly correlates with chronic kidney disease (CKD) and end-stage renal disease. For example, in the United States, approximately 22.4% of the end-stage kidney disease cases come from Medicare beneficiaries with a history of hospital-acquired AKI [3].

AKI is a syndrome characterized by a sudden loss of renal function associated with tubular injury, inflammation, and impaired renal blood flow [4]. The leading causes of AKI include ischemia–reperfusion injury, frequently occurring secondary to cardiopulmonary bypass surgery or transplant, sepsis, or exposure to nephrotoxic agents [4]. In addition, AKI is associated with progression of CKD, which is thought to result from inefficient tubular repair, sustained inflammation, and hypoxia due to capillary rarefaction [4, 5]. The development of new therapies to facilitate rapid and efficient renal repair is an important therapeutic goal to ease the burden of AKI.

Mesenchymal stem cells (MSC) isolated from bone marrow and adipose tissue (ASC) have been used in a variety of clinical and preclinical studies and have demonstrated beneficial anti-inflammatory effects and promoted vascular repair and functional recovery from ischemic injury [6–10]. As such, these cell-based therapies have received significant attention as a potential means to treat AKI [6, 7, 11–14]. Our lab has demonstrated that suprarenal aortic injection of human ASCs immediately following renal I/R injury protected rats against loss of renal function and vascular dropout [15]. However, our study and others [12, 16, 17] have shown that MSC or ASCs do not home to injured tissue, suggesting that the reparative effects of these cells are due to release of secreted factors.

Recently, the secretome of ASCs, a set of proteins and/or associated extracellular vesicles, released by cells into extracellular space, has received attention for the treatment of kidney injury [18, 19]. Importantly, most of these studies have primarily focused on injury prevention rather than recovery and repair [17, 20–22]. Whether systemic delivery of ASC-secreted factors improve recovery in established AKI is not known. The current study sought to determine whether cell-free therapy using ASC-derived secretome, generated using a scalable manufacturing process compliant with requirements to translate to production under current Good Manufacturing Practice, facilitates recovery following the establishment of AKI in a rat model of IRI.

## Methods

The work has been reported in line with ARRIVE 2.0 guidelines.

### ASC secretome generation and characterization

The T-101 test article in this study represents the concentrated secretome (CS) fraction of conditioned media from adult adipose stromal cells as described in a previous report [23] and will be referred to as ASC-CS for consistency. ASCs for the production of CS were derived from lipoaspirate collected under an IRB-approved protocol with informed consent from an adult healthy female donor that underwent elective lipoplasty. A master cell bank of ASC was produced in compliance with current Good Manufacturing Practice at Cytovance Biologics (Oklahoma City, OK) and tested for adventitious agents at BioReliance (Rockville, MD). Cell vials from the master cell bank were transported to Theratome Bio (Indianapolis, IN) in the vapor phase of LN2. ASC-CS was produced at Theratome Bio. All procedures were carried out in sterile, single-use containers under aseptic conditions. ASC were expanded in proprietary growth media (*passage 6–9*) in Corning Hyperflask culture vessels. At approximately 70% confluence, cells were washed 3 times with sterile cell culture grade PBS; serum free DMEM without phenol red was then added for conditioning. Cell expansion and media conditioning were carried out in a humidified cell culture incubator at 37 °C and 5% CO_2_. Conditioned media were collected after 60 h, centrifuged at 400 × g for 10 min to remove detached cells and debris, and then filtered through a 0.45 µM PES filter. Media was then concentrated (approximately 100 × volume reduction) using tangential flow filtration (3 kDa, PES filter membrane), buffer was exchanged 7 times with sterile, cell culture grade PBS, passed through 0.22 µM PES sterile filter, and then aliquoted in sterile, polypropylene cryovials. Vials with ASC-CS were stored at -70 to -90 °C and thawed immediately prior to use. Protein concentration was determined by Bradford assay. The profile of cytokines and growth factors in ASC-CS was analyzed using a Meso Scale Discovery multiplex assay (Rockville, MD USA).

### Animals, surgery, and study design

The experiments were performed in accordance with the policies of the National Institutes of Health *Guide for the Care and Use of Laboratory Animals* for Scientific Purposes. Experiments were approved by the Institutional Animal Care and Use Committee at Indiana University. Male Sprague Dawley rats (250—300 g) were purchased from Envigo (Indianapolis, IN). Rats were housed in pairs in the Indiana University Laboratory Animal Resource center with 12 h light cycle with food and water available ad libitum. All surgeries were performed between 0900 and 1300. As per IACUC guidelines, rats were monitored daily after surgery and euthanasia is indicated for rats based on a scoring system to evaluate excessive distress including the presence of excessive weight loss, lethargy, and loss of thermoregulation.

Rats were anesthetized by intraperitoneal injection of ketamine (100 mg/kg) and xylazine (5 mg/kg) and then placed on a heated pad to facilitate maintenance of body temperature. Anesthetized rats were subjected to bilateral renal ischemia–reperfusion I/R injury by clamping both left and right renal pedicle for 40 min to induce AKI, as described previously [15]. Sham-operated control rats were subjected to anesthesia and midline incision, but the kidneys were not touched. Rats were provided buprenorphine-SR (1 mg/kg) as analgesia.

Blood (~ 250 µl) was collected from the tail into heparinized-Eppendorf tubes and centrifuged at 3000 × g for 10 min. Plasma creatinine was quantified daily using a Point Scientific QT 180 Analyzer (Point Scientific Inc, Canton, MI, USA) and Angiopoietin-2 was evaluated on day 5 post IR using Angiopoietin-2 Quantikine ELISA Kit (Catalog #: MANG20**,** R&D Systems, Inc**.** MN). Rat plasma KIM-1 was measured using an assay from Mesoscale Discovery (Rockville, MD; R-PLEX Rat HAVCR1/KIM-1, catalog K1534UR-2).

**Fig. 1:**
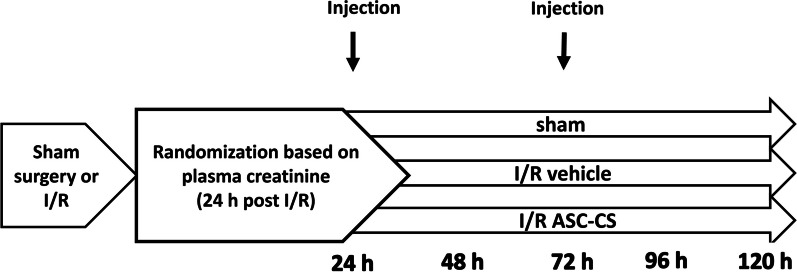
Experimental schema and timeline. Rats were anesthetized and subjected to bilateral renal ischemia–reperfusion (I/R) injury or sham surgery. At 24 h post-surgery, rats were randomized into two groups based on creatinine values such that the vehicle-control and treatment groups had similar levels of renal injury at the initiation of treatment. Rats were randomized into groups receiving subcutaneous injection of either ASC-CS (2 mg/kg; 1 ml) or saline-vehicle (1 ml) at 24 h and 72 h post I/R injury. After 5 days (120 h) recovery post-surgery, kidneys were harvested for histological evaluation and flow cytometry analysis of inflammatory cells

At 24 h post-surgery, rats were randomized into two groups based on creatinine values such that the vehicle-control and treatment groups had similar levels of renal injury at the initiation of treatment (Fig. 1). Of 31 rats subjected to IRI, one had an extremely high 24-h creatinine value of 5.7 mg/dl and was excluded from the study, as rats with this degree of injury are not likely to survive for the duration of the experiment. All other rats were randomized into groups receiving subcutaneous injection of either ASC-CS (2 mg/kg; 1 ml) or saline-vehicle (1 ml). No rats in either group died or required euthanasia after randomization.

After 5 days recovery post-surgery (4 days post-treatment), animals were euthanized following deep isoflurane anesthesia and subsequent pneumo-thoracotomy and exsanguination. Kidneys were rapidly harvested for histological evaluation and flow cytometry analysis of inflammatory cells. For histology, portions of the kidney were fixed in 10% buffered formalin. A subset of 6 post-IRI rats (3 per group) were not euthanized on Day 5 and therefore were not included in analysis of tissue.

### Histology

Formalin-fixed paraffin-embedded kidney sections were stained with periodic acid Schiff and imaged with Leica EC4 camera (Scientific Instruments, 176 Columbus, OH) mounted on a Nikon Optiphot-2 microscope. Renal injury was scored using an approach previously described by our group [24]. Briefly, 5 random images through the renal outer medulla were scored with the aid of ImageJ software (U.S. National Institutes of Health, Bethesda, MD, USA) by a blinded observer. Individual tubules were categorized as either undamaged, or showing moderate-damage or severe damage, where severely damage tubules contained abundant cellular debris and a thinned epithelial layer; moderately damaged tubules had minimal cellular debris and a more hypertrophied epithelium. Data are expressed as percent of tubules with severe damage.

### Flow Cytometry analysis of inflammatory cells

Analysis of inflammatory cells was based on previously described methods [15]. Briefly, after kidney tissue digestion (2 µg/ml liberase, Roche, Indianapolis, IN) and Percoll (Sigma, St. Louis, MO) separation, mononuclear cells were stained for flow cytometry analysis. T lymphocytes were identified with anti-rat antibodies against CD4 (PE-Cy7; BD Bioscience #561,578), CD8a (Alexa 647; BD 561611), FOXp3 (PE; eBioscience #12 5773 82) and CD25. For identification of IL-17 secreting capacity, cells were stained for surface CD4, then permeabilized with 0.1% saponin and stained with antibodies against rat IL-17 (FITC; eBioscience #11 7177 80). Macrophage/dendritic cells were identified using antibody against CD11b/c (BD Bioscience #554,862). Labeled cells were analyzed using BD LSRFortessa analyzer (BD Biosciences, San Jose, CA, USA) and Flowjo software (Tree Star, Ashland, OR, USA). Gating strategies were followed as described previously [25] and are shown in Additional file 1: Fig. S1. Data were analyzed by a member blinded to treatment groups.

#### In vitro* Th17 activation*

In vitro activation of kidney injury primed lymphocytes was based on methods previously described [26]. Briefly, CD4 + lymphocytes were isolated using magnetic separation (Miltenyi CD4 anti-rat microbeads; #130–090-319) from kidneys 7 days following recovery from IRI, at which time IL17A expression levels are typically restored to sham control levels. Cells (~ 250,000 per well in 48 well plate) plated CD3-coated plates with soluble anti-CD28. Cells were then incubated overnight at 37 °C in RPMI media supplemented with 10% FBS (Invitrogen), with some stimulated with elevated extracellular sodium (to 170 mM) and Ang II (Millipore Sigma, 10^−7^ M) with or without ASC-CS (adjusted to a final concentration of 2.5 µg/ml protein). During the final four hours of incubation, cells were incubated with 8 µM monensin (Golgistop, 1 μg/mL; BD Biosciences). Harvested cells were then stained for IL17A and analyzed by FACS.

#### Statistical analysis

Data are presented as mean ± standard error of the mean (SEM). Statistical analysis was performed with GraphPad prism 9.2 (GraphPad Software, La Jolla, CA). Variables measured at multiple time-points were analyzed using repeated measures analysis of variance (ANOVA). To protect against increased risk of type 1 error from the use of multiple comparisons Tukey’s test was applied for possible comparisons between groups. Student’s unpaired t-test was used for dichotomous comparisons. Two-sided P ≤ 0.05 was considered statistically significant..

## Results

### Characterization of ASC secretome

The ASC-CS from conditioned media of human ASCs has been described by us previously [23] and was prepared at a concentration of 0.6 mg total protein/ml. Since the effects of ASC-CS are attributed to multiple factors that influence cell function, assays for cytokines and growth factors are routinely conducted for selected factors to ensure batch-to-batch ASC-CS consistency. As a measure of quality control and consistency, values for various selected cytokines in ASC-CS batch used in this study are found in Table 1, which is characterized by high concentrations of VEGF, HGF and IL-8 as well as other cytokines, and is consistent with the composition of T101/ASC-CS routinely generated by Theratome using the procedure described.

**Table 1:** Characterization of adipose derived secretome using Mesoscale multiplex assay platform

Cytokines	pg/μg of protein
Vascular endothelial growth factor	212.2 ± 6.8
*Hepatocyte Growth Factor*	181.6 ± 2.4
Interleukin 4	0.61 ± .02
Interleukin 6	48.7 ± 1.7
Interleukin 8	287.0 ± 0.5
Interleukin 10	0.16 ± 0.01
Brain-derived neurotrophic factor	2.28 ± 0.02

Data represent mean ± SEM. Assays were performed on duplicate samples

### ASC-CS improves renal function following bilateral renal IRI

**Fig. 2:**
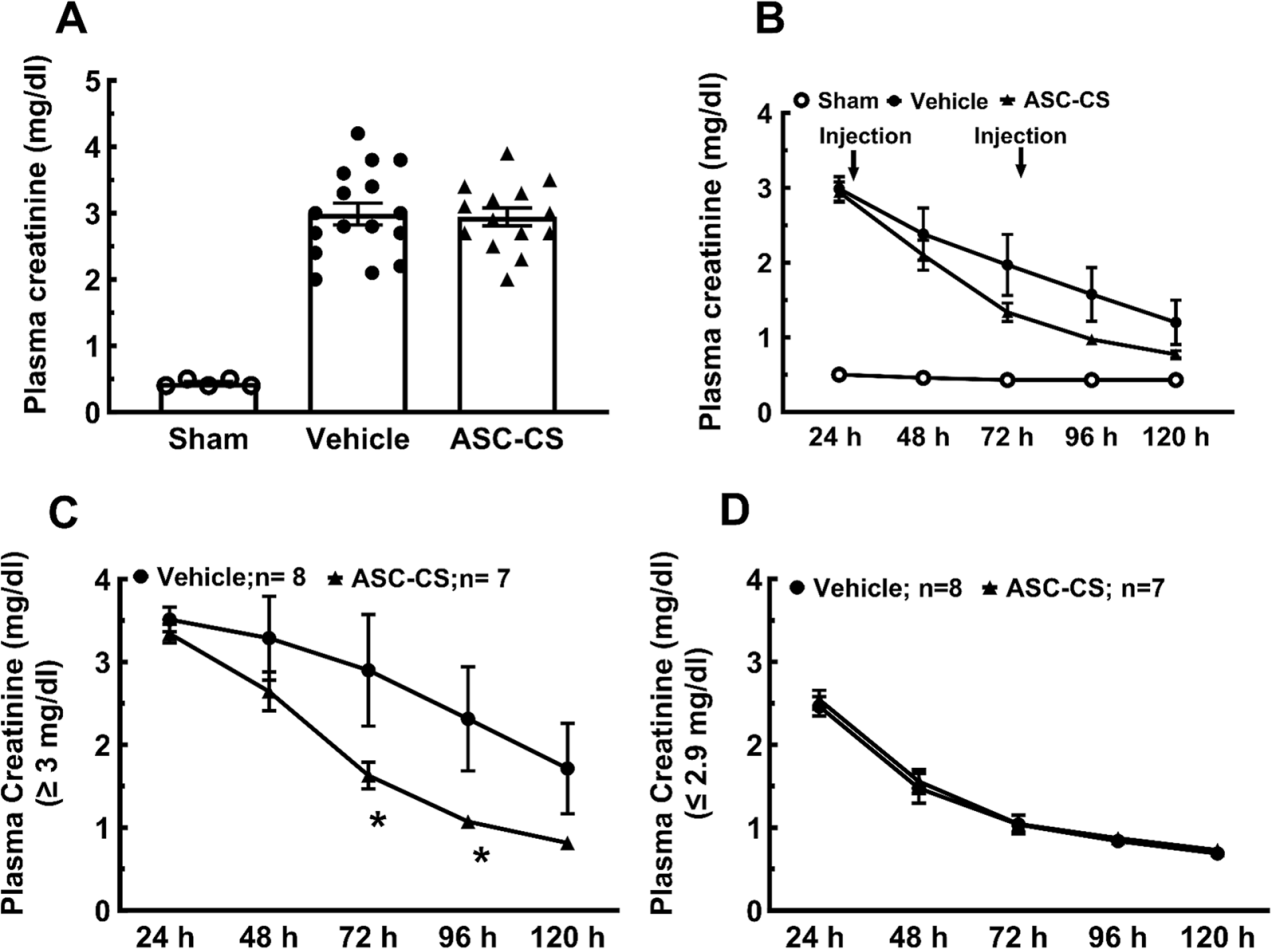
Secretome improves renal function following bilateral I/R injury. Plasma creatinine levels were measured longitudinally during a 5 day protocol following either sham surgery or post IRI injury. **A** Plasma creatinine 24 h post I/R prior to randomization of rats into respective treatments (Sham: *n* = 7; Vehicle: *n* = 16; and ASC-CS: *n* = 14) (B) Rats received two injections of either secretome (2 mg/kg; 1 ml) or saline (vehicle; 1 ml) via subcutaneous injection after 24 h and 72 h post IRI (arrows) following collection of blood for renal functional assessment. Change of plasma creatinine levels over time in rats with initial creatinine values of 3.0 mg/dl or greater (C) or in rats with creatinine values 2.9 mg/dl or lower (D). Data are presented as mean ± SEM. *indicates *P* < 0.05 vs in ASC-CS vs vehicle treated animals by repeated measures ANOVA and Tukey’s post-hoc test

The plasma creatinine values of rats 24 h post IRI prior to treatment is shown in Fig. 2A; creatinine values were similar between groups indicating a successfully balanced randomization (2.9 ± 0.2 mg/dl for I/R vehicle vs 2.9 ± 0.1 mg/dl for I/R ASC-CS; P = 0.83, unpaired *t* test). Within 1 day of treatment with ASC-CS or vehicle treatment (at 48 h post I/R), plasma creatinine levels decreased relative to the 24 h time point and continued to decline thereafter. While the decline in creatinine tended to be faster in the ASC-CS group, there was no significant difference between ASC-CS and vehicle (Fig. 2B).

Although the randomization strategy appeared effective, the injury level following I/R was variable, as is typical with this model, with 24-h creatinine values ranging between 2.0 and 4.2 mg/dl (Fig. 2A). Based on our experience, rats on the lower end of this range are expected to have a more rapid and complete recovery versus rats on the high end of this range. To gain further insight into the potential effect of ASC-CS on recovery from established AKI, we subdivided rats into upper or lower halves of AKI severity based on the distribution of 24-h creatinine values in this study prior to treatment. The upper half of creatinine values were ≥ 3.0 mg/dl (referred to as severe injury), while the lower half was ≤ 2.9 mg/dl or lower (referred to as moderate injury). The rates of recovery of renal function in each of these subgroups are shown in Fig. 2C and 2D, respectively. In rats with severe injury, ASC-CS significantly reduced plasma creatinine within 24 h of treatment vs vehicle, to values that were not different from sham within 5 days. In contrast, in vehicle-treated rats from the severe injury group, creatinine values remained elevated relative to sham for at least 5 days indicative of less efficient recovery (Fig. 2C). Rats with moderate injury recovered rapidly toward sham-control values, regardless of whether these were treated with ASC-CS or vehicle (Fig. 2D), suggesting that ASC-CS mitigates severe AKI, but there is little dynamic range to provide an effect on recovery after moderate AKI.

**Fig. 3:**
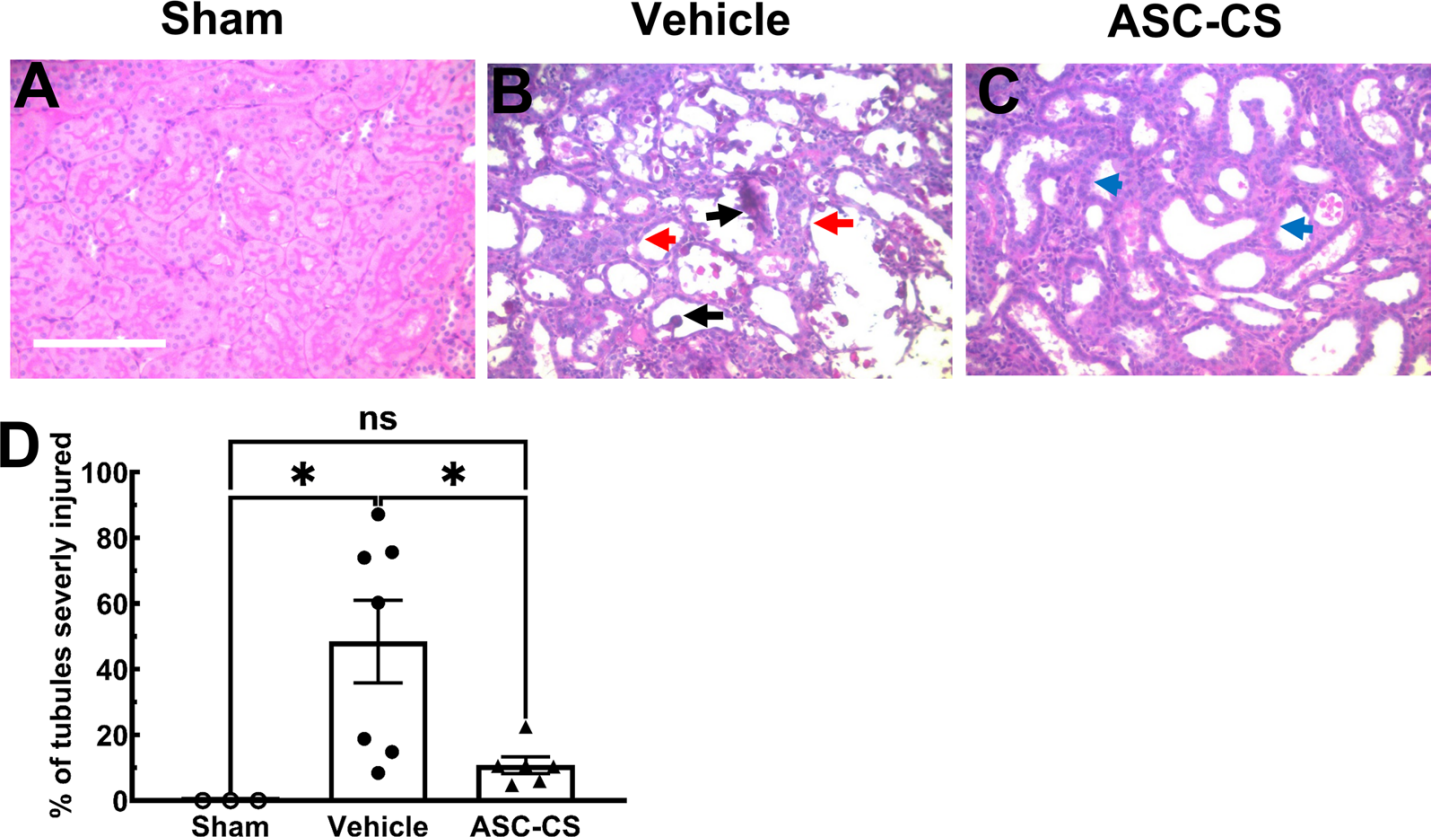
Secretome improved kidney structure following bilateral I/R injury. Representative micrographs of PAS stained sections of outer medulla from sham **A**, vehicle **B** and ASC-CS **C** treated rats are shown 5 days following I/R. Evident tubular damage with abundant cellular debris in the lumen (black arrows), simplified cells lining the basement membrane (red arrow) are frequently observed in vehicle-treated rats **B**. Minimal tubule debris and a relatively greater degree of hypertrophy was observed regenerating tubules of ASC-CS treated rats (blue arrow); Bar in panel A is 200 µm. **D** % of severely injured tubules. Data correspond to rats with the greatest degree of initial injury (SCr ≥ 3.0) and are presented as mean ± SEM. *P < 0.05, by Tukey’s multiple comparison test

Based on the finding that ASC-CS has evident beneficial effects in more severe AKI (creatinine > 3.0 mg/dl at 24 h post IRI), analysis of renal structure was performed on the tissues of the rats in this group. Figure 3 shows representative micrographs of PAS-stained sections of renal outer medulla, the region of the kidney where the most significant tubular damage is observed [4]. After 5 days of recovery from I/R, vehicle-treated rats continued show evident tubular damage, with abundant cellular debris in the lumen relative to sham (Fig. 3A** vs 3B**; black arrows) as well as abundant small, simplified cells lining the basement membrane (red arrow). In contrast, ASC-CS rats manifested improved recovery of tubules, with minimal tubule debris and relatively hypertrophied regenerating tubules vs vehicle (Fig. 3C; blue arrow). The percentage of severely damaged tubules was significantly decreased in ASC-CS rats vs vehicle treated rats (Fig. 3D). As a further assessment of renal tubular injury, circulating KIM-1 levels were measured in plasma samples. While KIM-1 levels were highest in the severe injury-vehicle group, ASC-CS treatment significantly attenuated plasma KIM-1 (Additional file 1: Fig. S2).

### ASC-CS reduces infiltration of inflammatory cells and attenuates angiopoietin -2 levels

**Fig. 4:**
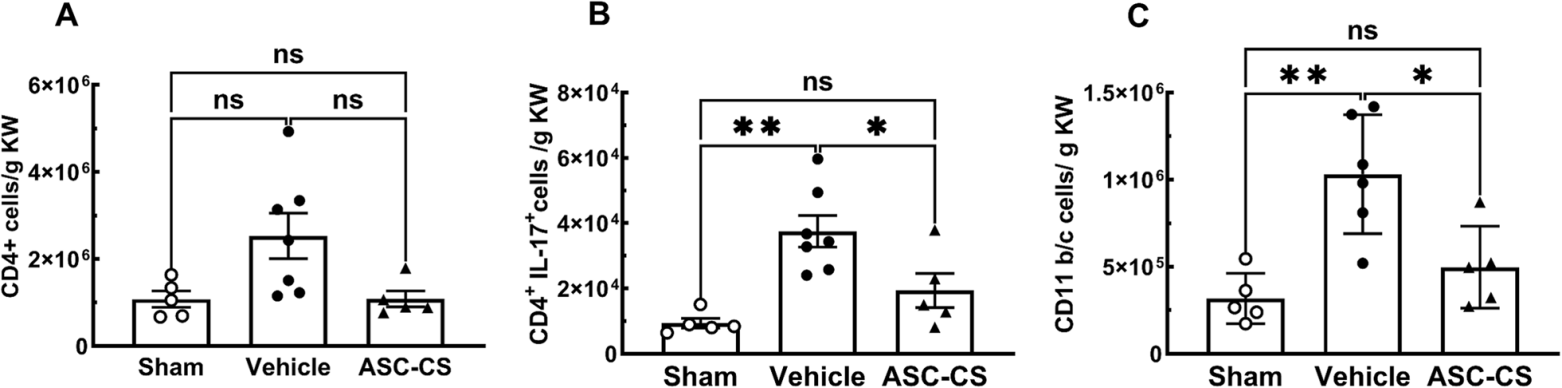
Secretome reduces renal accumulation of CD4 + IL17 cells and CD 11/b/c following I/R injury. Kidney-resident lymphocytes were isolated after 5 days post IRI. **A** CD4 + cells **B** CD4 + IL17 + cells and **C** CD11b/c cells. Data correspond to rats with the greatest degree of initial injury (PCr ≥ 3.0) and are expressed as number of cells per gram of kidney weight. Values are mean ± SEM. *indicates *P* < 0.05 and ** indicates *p* < 0.01 by Tukey’s multiple comparison test

To evaluate the potential anti-inflammatory effects of ASC-CS, we enumerated infiltration of immune/inflammatory cells into kidney of post-I/R rats. In the severe injury group, total CD4 + lymphocytes tended to be increased but were not significantly elevated above sham (Fig. 4A) while Th17 cells (CD4 + IL-17 + , Fig. 4B) were elevated versus sham and were significantly reduced versus vehicle-treated controls. Similarly, IR injury significantly increased the number of CD11b/c (dendritic/macrophage) cells in the vehicle-treated group relative to sham, and their accumulation was also reduced by ASC-CS treatment (Fig. 4C**).** ASC-CS also tended to reduce accumulation of CD8 +, CD8 + IL17 + and CD4 + Foxp3 + cells, but the differences were not statistically significant (Table 2). Similar effects of ASC-CS on inflammatory cell infiltration were observed when both severe and moderate injury groups were included in the analysis (Additional file 1: Fig. S3 and Table S1).

**Table 2:** Effects of secretome on infiltration of cells after 5 days following renal I/R injury

	Sham	Vehicle	ASC-CS
Total mononuclear cells	3.8 ± 0.7 × 10^6^	7.2 ± 1.2 × 10^6^	3.7 ± 0.6 × 10^6^
CD8 +	3.0 ± 0.3 × 10^4^	6.6 ± 0.2 × 10^5^*	2.9 ± 0.9 × 10^5^
CD8 + IL17 +	6.2 ± 0.8 × 10^2^	1.9 ± 0.4 × 10^3^	2.1 ± 0.8 × 10^3^
CD4 + Foxp3 +	1.1 ± 0.2 × 10^4^	2.3 ± 0.3 × 10^4^*	1.6 ± 0.3 × 10^4^
Foxp3 (%)	0.4 ± 0.1	1.5 ± 0.2	2.1 ± 0.7 †

Values are expressed in mean ± SEM and derived from rats in the severe injury group (PCr ≥ 3.0 mg/dl). Inflammatory cell types are expressed per gram of kidney weight. *indicates *P* < 0.05 Sham vs Vehicle, and ^†^ indicates *P* < 0.01 Sham vs ASC-CS by Tukey’s multiple comparison test

**Fig. 5:**
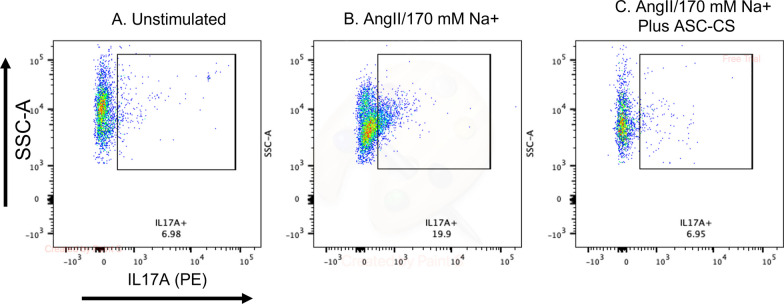
ASC-CS inhibits IL17 activation in AKI primed lymphocytes in vitro. Representative FACS showing increased IL17A expression in isolated CD4 + cells from kidneys of 7-day post I/R injury rats incubated overnight under **A** control conditions, **B** stimulated with Ang II and 170 mM Na + or (C) Ang II and 170 mM Na + with ASC CS **C**

The decrease in inflammation may result from less severe tissue damage that triggers the inflammatory response and may also be due to a direct effect on the inflammatory cells. To address whether the latter represents a possible mechanism, we used an assay established by our group in which CD4 + lymphocytes isolated from rat kidney (7 days post I/R) re-express IL17A in response to Ang II and elevated Na^+^ [26, 27]. Stimulation of injury-primed lymphocytes (~ 16 h) with AngII/Na^+^ increased the prevalence of IL17A-expressing cells relative to unstimulated lymphocytes (Fig. 5A, B), but ASC-CS treatment of AngII/Na^+^ stimulated cells ablated this effect (Fig. 5C).

**Fig. 6:**
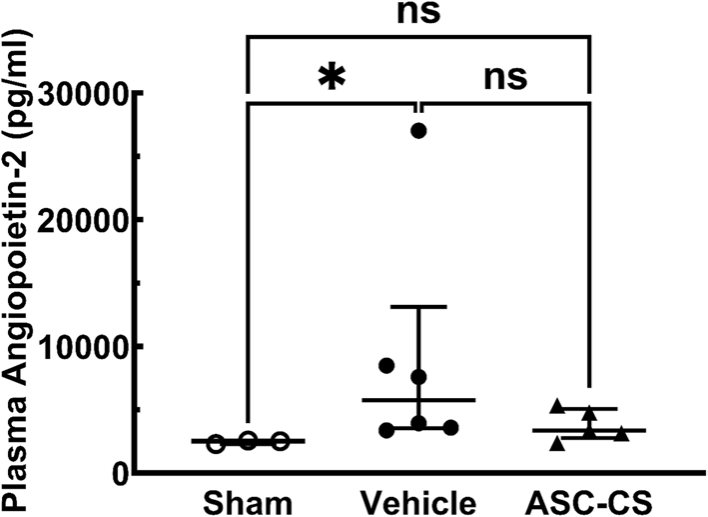
ASC-CS reduces plasma angiopoietin-2 level after 5 days post I/R injury. Plasma angiopoietin was assessed by ELISA in plasma collected from rats with serum creatinine ≥ 3.0 mg/dl. Data correspond to rats with the greatest degree of initial injury (SCr ≥ 3.0) and are presented as mean ± SEM. *indicates *P* < 0.05 Tukey’s multiple comparison test

ASC-CS may also have vascular protective effects. Angiopoietin-2 (Ang-2) can be released by damaged or inflamed endothelial cells and has been associated with vascular injury and worsened outcomes in AKI [28, 29]. Within the severe-injury subgroup of rats, a significant increase in plasma Ang-2 was detected in vehicle-treated rats relative to sham control rats at Day 5 post I/R, while Ang-2 levels were not different from sham in ASC-CS treated post-ischemic rats (Fig. 6**).** Within the moderate injury group, Ang-2 levels were not different from sham (not shown).

## Discussion

AKI is characterized by an abrupt and frequently irreversible loss of excretory kidney function and represents a major health concern in hospitalized patients worldwide that is associated with substantial morbidity and mortality. While many surviving patients may recover renal function to pre-injury levels, AKI is a major risk factor for the development of CKD or end stage renal disease, adding to the clinical burden of this disorder [30, 31]. The pathophysiology of AKI is complex, and the majority of therapeutic measures are supportive modalities. In some cases, treatments intended to avoid ongoing damage following AKI have met with disappointing results [32], and effective therapeutic options for promoting kidney regeneration or repair following AKI remain scarce.

Over the past few decades, there has been much interest revolving around cell-based therapies for application in regenerative medicine and cell-based strategies have shown the potential to affect the course of AKI in pre-clinical models. This includes seminal studies by Lange et al., demonstrating that systemic administration of bone marrow-derived MSCs to post-ischemic rats significantly improved kidney excretory function at days 2 and 3 after cell therapy, increased tubular epithelial cell proliferation, decreased apoptosis and tissue damage [13]. Similar reno-protective effects of MSCs have been described in several other reports [6, 7, 11–14]. It has been suggested that the MSC-derived protective activity is due to the release of a variety of released soluble factors, including VEGF [33].

ASCs are a type of MSC that have several advantages relative to bone marrow-derived MSC, as they are abundant in the adult human body and relatively easy to obtain. Multiple studies, including our prior study, have demonstrated the efficacy of hASCs in preclinical models of AKI, in which they improve renal function, reduce tissue inflammation, enhance tubular repair, preserve vascular integrity, and lower mortality [14, 15].

The mechanism of MSC/ASC-mediated protection is not fully understood. Originally, considered as a source of adult progenitor cells to mediate tissue repair, there are discrepant reports that injected MSCs home to the site of tissue injury [11–13, 34]. In our own study, suprarenal injection of ASCs preserved kidney function in the absence of homing [15], suggesting that the therapeutic effect is mediated by factors liberated from ASC and/or modulation of distant cells or tissues, such as cells comprising the immune system. Indeed, numerous recent reports describe soluble factors in conditioned media or in media-derived exosomes that promote vascular repair or inhibit inflammation [9, 19]. These activities are important as one considers the potential application of ASCs to the point-of-care for patients in the ICU with AKI. We suggest that the therapeutic use of ASC secretome offers both logistical and potential safety advantages versus direct administration of cells.

The timing of treatment initiation in pre-clinical models should seek to represent real-world scenarios to assess the potential efficiency of a therapy for AKI. It is likely that significant tissue injury is already present by the time the diagnosis of AKI is made [35]. For this reason, treatments should focus on recovery following the establishment of AKI. In the current study, significant injury was established 24 h post-IRI. In rats, this time-point is associated with severe tubular damage via multiple forms of cell death, impaired vascular function, including vasoconstriction and vascular and tubular congestion, and a fall in GFR. Depending upon the severity of the initial injury in rat I/R models, recovery of serum creatinine occurs over the course of 5–8 days, while tubular morphology may not be complete for several weeks [4]. Other aspects of renal structure and function may not be fully restored; for example, a population of tubular cells may assume a novel phenotype associated with a failed repair response, and there is evidence of persistent inflammation and permanent vascular rarefaction [4, 5, 31]. All of these have been proposed to play an important role in the transition of AKI to subsequent CKD and fibrosis, and all of these may benefit from trophic, angiogenic and anti-inflammatory activity from ASC-derived secretome.

The current report contains several unique elements highlighting the potential utility of ASC-CS in the setting of AKI: 1) Unlike previous reports using intravenous administrations of cells, there was a rapid response of improved renal function noticeable within 1 day following a single-subcutaneous injection of ASC-CS. Given that many effects of ASC are ascribed to Secreted factors, it is likely cells, which effectively represent prodrugs relative to secretome, may have delayed onset of action versus secretome. In addition, the subcutaneous route of ASC-CS administration was selected based on the recent report by Bogatcheva et al., which demonstrated the utility of a similar approach to alleviate lung inflammation in a model of influenza in mice [23]. This approach may have pharmacokinetic and/or biodistribution properties favorable for the observed effects. Although a second injection was provided 48 h after the first injection, the temporal trajectory of recovery suggests that the recovery was predominantly due to the effects of the initial injection. 2) As described before, the model used for randomization was chosen to avoid any potential imbalance in the severity of AKI between the treatment groups and was geared to address recovery from AKI rather than initiation. 3) Because our sample size was sufficient to subdivide into groups based on injury severity, we were able to determine that ASC-CS was effective in improving recovery in the most severely injured subset of rats. As rats from the lower half of the creatinine range have less severe tissue damage, the more rapid recovery and lack of additional therapeutic benefit in this group of rats should not be surprising.

ASCs can release multiple growth factors, chemokines, cytokines, and extracellular vesicles. For example, trophic factors such as vascular endothelial growth factor (VEGF), basic fibroblast growth factor (bFGF), insulin-like growth factor 1 (IGF-1), platelet-derived growth factor (PDGF), hepatocyte growth factors (HGF), and transforming growth factor β1 (TGF-β1) have been recognized as the main mediators of the pro-angiogenic, immunomodulating, and anti-apoptotic effects of ASCs [36]. The ASC-CS generated under conditions described here also contains large amounts of VEGF and IL-8. While the characterization of secretome is important to evaluate consistency for production, and may provide clues to mechanism, it is important to point out that the specific factor or factors that mediate the protective effects reported here are uncertain. Indeed, the beneficial effects are likely mediated via combined activity by multiple paracrine factors [37, 38].

The reduction of Th17 cells reported here is consistent with our previous report using an intra-arterial injection of ASCs at the time of reperfusion [15]. Th17 cells are the most abundant lymphocyte population induced in rat kidney following I/R [27], and inhibition of its primary secreted cytokine IL17 protects against early acute injury and CKD progression [25]. In the current report, we demonstrate that ASC-CS reduced in vitro activation of lymphocytes primed by injury to express the Th17 phenotype. To the best of our knowledge, this is the first demonstration that ASC-secretome may directly inhibit lymphocyte activation to the Th17 phenotype.

In our previous report, we also demonstrated that human ASCs injected intra-arterially protected against the loss of peritubular capillaries following AKI [15]. It is possible that ASC-CS also has a similar protective effect on vascular damage since Ang-2 levels, which have been shown to correlate with vascular rarefaction [28, 29], were attenuated by ASC-CS. Unfortunately, in the current study, there was a limited availability of appropriately fixed tissues that prevented us from directly measuring capillary density.

Despite our attempts to minimize bias by performing randomization based on a quantitative assessment of renal function prior to treatment initiation, the rat model contains several inherent weaknesses. Included among these whether surgery- induced AKI in young healthy rats recapitulates elements of recovery in critically ill human patients with AKI. In addition, the current study did not assess long term function and structure at later time points to determine if the treatments may mitigate the development of chronic kidney disease, fibrosis, and hypertension frequently reported following recovery from AKI [4, 5, 31].

## Conclusions

AKI remains a significant clinical concern with no available treatment options. Cell-based therapies have garnered significant attention in recent years, but the lack of homing and significant logistical considerations delivering cellular material to an acute care setting present significant obstacles to therapy [39]. Moreover, a general failure of preclinical studies to focus on recovery has hampered the field. The current report demonstrates that concentrated secretome of human ASC is a significant step toward accelerating the pursuit of cell-derived therapies in AKI. Given that ASC and other mesenchymal stromal cells have already been widely used with good safety profile [40, 41], this study may help guide a pathway for the use of ASC-secretome in AKI patients.

### Supplementary Information

**Additional file1**: **Fig. 1**. Representative gating strategy for characterization of mononuclear cell composition in rat kidney. Percoll separated cells were stained as described in methods. Initial analysis of FSc and SSc identified single mononuclear cells for additional analysis as shown. CD4+ cells or CD11b/c cells were identified based on gates established using FMO. Th17 cells were identified as IL17A expressing cells from the CD4+ population as indicated. **Fig. 2**. Circulating KIM-1 levels are reduced following treatment with ASC-CS. Shown are KIM-1 levels in sham, AKI-vehicle and AKI-CS rats 5 days following Surgery. Values are mean± SEM. * indicates *P*<0.05 AKI-vehicle vs AKI-ASC-CS and ** indicates *P*<0.01 sham vs AKI-vehicle, by ANOVA and Tukey’s multiple comparison test. **Fig. 3**. Secretome reduces renal accumulation of CD4+IL17 cells and CD 11/b/c following IRI. Kidney resident lymphocytes were isolated after 5 days post IRI. (A) CD4+ cells (B) CD4+IL17+ cells and (C) CD11b/c cells. Data correspond to all rats categorized as severe and moderate initial injury and are expressed as number of cells per gram of kidney weight. Values are mean± SEM. *indicates *P*<0.05 and ** indicates *p*<0.01 by Tukey’s multiple comparison test. **Table 1**. Effects of ASC-CS on infiltration of cells after 5 days following renal I/R injury

## Data Availability

The data that support the findings of this study are available from the corresponding author upon reasonable request.

## Abbreviations

AKI: Acute kidney injury
Ang II: Angiotensin II
Ang-2: Angiopoietin-2
ASC: Adipocyte stromal cell
bFGF: Basic FGF
CKD: Chronic kidney disease
CS: Concentrated secretome
GFR: Glomerular filtration rate
HGF: Hepatocyte growth factor
IL8, IL17: Interleukin-8, interleukin-17
I/R: Ischemia/reperfusion
MSC: Mesenchymal stem cells
PAS: Periodic acid Schiff
PBS: Phosphate buffered saline
Pcr: Plasma creatinine
TGF-β: Transforming growth factor-β
Th1, Th2, Th17: T-helper 1, T-helper 2, T-helper 17
VEGF: Vascular endothelial growth factor
