# Supplementary material for: Subcutaneous injection of adipose stromal cell-secretome improves renal function and reduces inflammation in established acute kidney injury
Source: Stem Cell Res Ther. 2024 Apr 24;15:119. doi: 10.1186/s13287-024-03736-x (PMC11040889; doi:10.1186/s13287-024-03736-x)
Supplement: Supplementary file 1 — Additional file1: Fig. 1. Representative gating strategy for characterization of mononuclear cell composition in rat kidney. Percoll separated cells were stained as described in methods. Initial analysis of FSc and SSc identified single mononuclear cells for additional analysis as shown. CD4+ cells or CD11b/c cells were identified based on gates established using FMO. Th17 cells were identified as IL17A expressing cells from the CD4+ population as indicated. Fig. 2. Circulating KIM-1 levels are reduced following treatment with ASC-CS. Shown are KIM-1 levels in sham, AKI-vehicle and AKI-CS rats 5 days following Surgery. Values are mean± SEM. * indicates P<0.05 AKI-vehicle vs AKI-ASC-CS and ** indicates P<0.01 sham vs AKI-vehicle, by ANOVA and Tukey’s multiple comparison test. Fig. 3. Secretome reduces renal accumulation of CD4+IL17 cells and CD 11/b/c following IRI. Kidney resident lymphocytes were isolated after 5 days post IRI. (A) CD4+ cells (B) CD4+IL17+ cells and (C) CD11b/c cells. Data correspond to all rats categorized as severe and moderate initial injury and are expressed as number of cells per gram of kidney weight. Values are mean± SEM. *indicates P<0.05 and ** indicates p<0.01 by Tukey’s multiple comparison test. Table 1. Effects of ASC-CS on infiltration of cells after 5 days following renal I/R injury [file 13287_2024_3736_MOESM1_ESM.docx]

**Supplemental Figure 1: Representative gating strategy for characterization of mononuclear cell composition in rat kidney.** Percoll separated cells were stained as described in methods. Initial analysis of FSc and SSc identified single mononuclear cells for additional analysis as shown. CD4+ cells or CD11b/c cells were identified based on gates established using FMO. Th17 cells were identified as IL17A expressing cells from the CD4+ population as indicated.


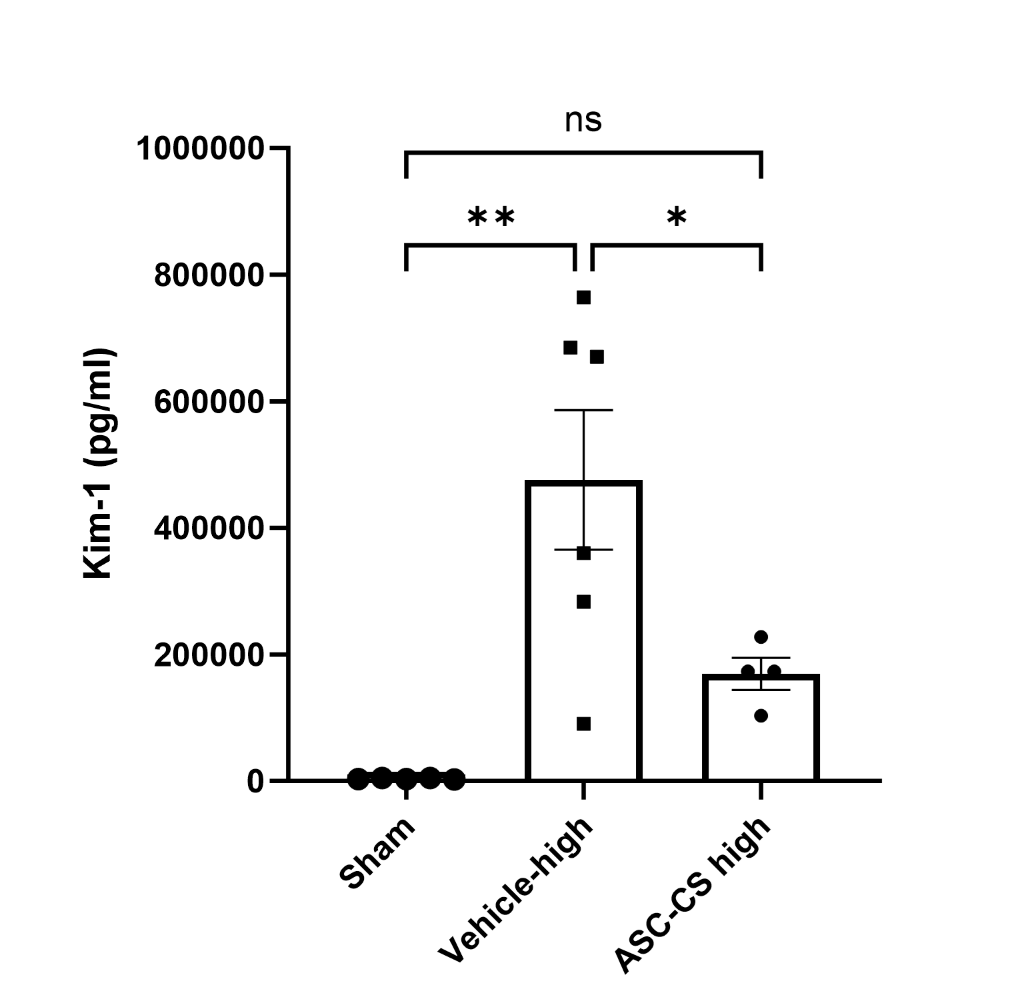


**Supplemental Figure 2:** Circulating KIM-1 levels are reduced following treatment with ASC-CS. Shown are KIM-1 levels in sham, AKI-vehicle and AKI-CS rats 5 days following surgery. Values are mean± SEM. * indicates P<0.05 AKI-vehicle vs AKI-ASC-CS and ** indicates P<0.01 sham vs AKI-vehicle, by ANOVA and Tukey’s multiple comparison test.


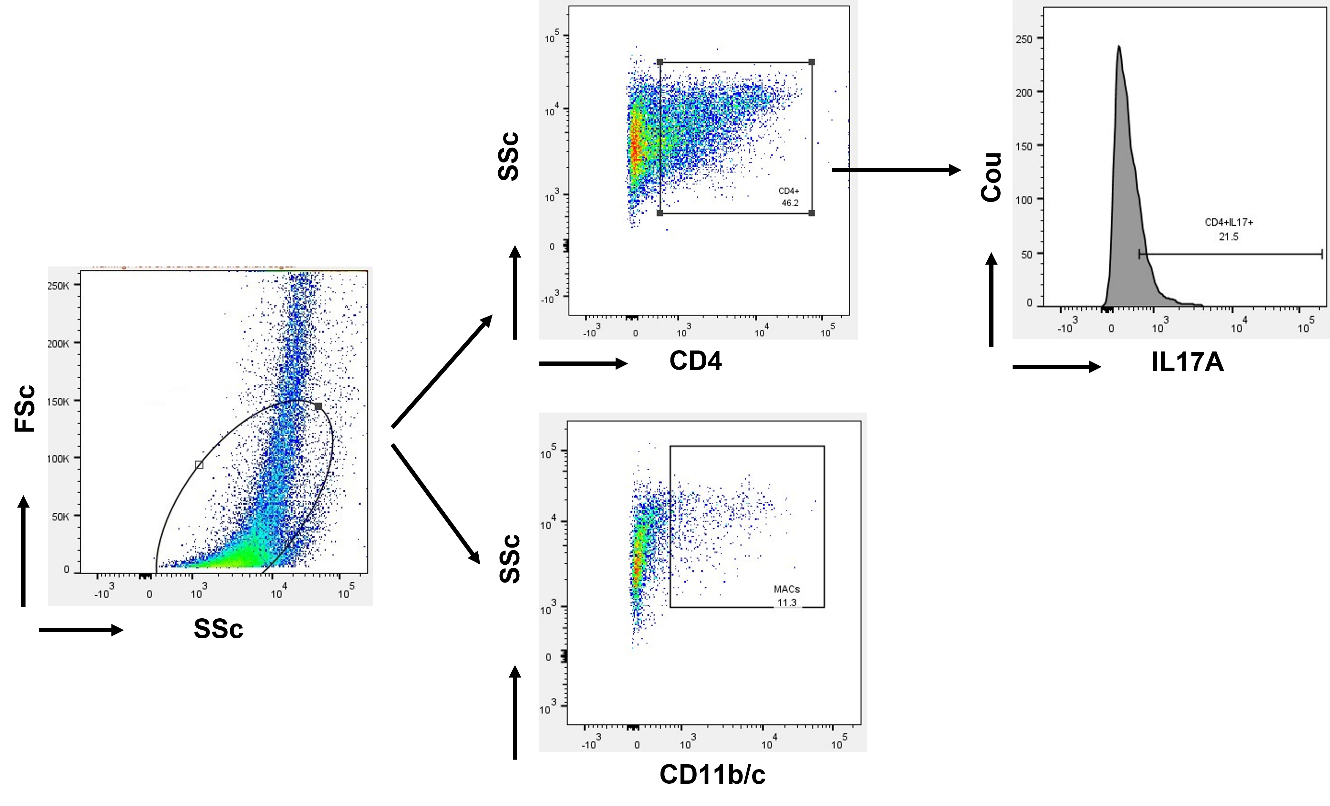


**Supplemental Figure 3: Secretome reduces renal accumulation of CD4+IL17 cells and CD 11/b/c following IRI**. Kidney resident lymphocytes were isolated after 5 days post IRI. (A) CD4+ cells (B) CD4+IL17+ cells and (C) CD11b/c cells. Data correspond to all rats categorized as severe and moderate initial injury and are expressed as number of cells per gram of kidney weight. Values are mean± SEM. *indicates P<0.05 and ** indicates p<0.01 by Tukey’s multiple comparison test.


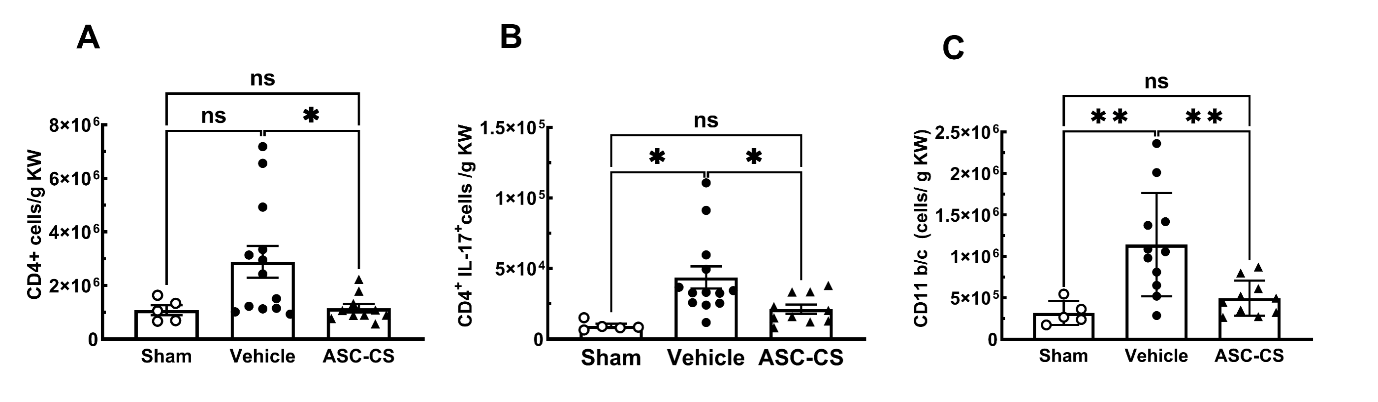


Supplemental Table 1: Effects of ASC-CS on infiltration of cells after 5 days following renal I/R injury

|  | Sham | Vehicle | ASC-CS |
| --- | --- | --- | --- |
| Total mononuclear cells | 3.9 ± 0.7×10^6^ | 8.6 ± 1.5×10^6^ | 3.9 ± 0.5 ×10^6^ **#** |
| CD8+ | 2.9 ± 0.3×10^4^ | 8.3± 1.9×10^5^* | 3.3 ± 0.8×10^5^ |
| CD8+IL17+ | 0.61 ± 0.1 ×10^3^ | 2.9± 0.7×10^3^ | 1.9 ± 0.5×10^3^ |
| CD4+Foxp3+ | 1.1± 0.2 ×10^4^ | 4.5 ± 1.3×10^4^ | 2.5± 0.8×10^4^ |
| Foxp3(%) | 0.4 ± 0.1 | 2.1 ± 0.3 | 2.4 ± 0.7 † |

Values are expressed in mean± SEM and reflect combined data obtained from both severe and moderate injury rats in the study. Inflammatory cell types are expressed per gram of kidney weight. *indicates P<0.05 Sham vs Vehicle, #indicates p<.05 Vehicle vs ASC-CS and ^†^ indicates P<0.01 Sham vs ASC-CS by Tukey’s multiple comparison test.
